# Anthracene detoxification by Laccases from indigenous fungal strains *Trichoderma lixii* FLU1 and *Talaromyces pinophilus* FLU12

**DOI:** 10.1007/s10532-024-10084-3

**Published:** 2024-06-01

**Authors:** Samson O. Egbewale, Ajit Kumar, Tosin A. Olasehinde, Mduduzi P. Mokoena, Ademola O. Olaniran

**Affiliations:** 1 Discipline of Microbiology, University of KwaZulu-Natal (Westville Campus), Durban, 4000 South Africa; 2 Department of Pathology, School of Medicine, University of Limpopo, Private Bag X1106, Sovenga, 0727 South Africa

**Keywords:** Alzheimer, Anthracene, ABTS, Laccase

## Abstract

The persistence and ubiquity of polycyclic aromatic hydrocarbons (PAHs) in the environment necessitate effective remediation strategies. Hence, this study investigated the potential of purified Laccases, *Tl*FLU1L and *Tp*FLU12L, from two indigenous fungi *Trichoderma lixii* FLU1 (*Tl*FLU1) and *Talaromyces pinophilus* FLU12 (*Tp*FLU12), respectively for the oxidation and detoxification of anthracene. Anthracene was degraded with *v*_max_ values of 3.51 ± 0.06 mg/L/h and 3.44 ± 0.06 mg/L/h, and *K*_m_ values of 173.2 ± 0.06 mg/L and 73.3 ± 0.07 mg/L by *Tl*FLU1L and *Tp*FLU12L, respectively. The addition of a mediator compound 2,2-azino-bis (3-ethylbenzothiazoline-6-sulfonic acid) (ABTS) to the reaction system significantly increased the degradation of anthracene, with up to a 2.9-fold increase in *v*_max_ value and up to threefold decrease in *K*_m_ values of *Tl*FLU1L and *Tp*FLU12L. The GC–MS analysis of the metabolites suggests that anthracene degradation follows one new pathway unique to the ABTS system—hydroxylation and carboxylation of C-1 and C-2 position of anthracene to form 3-hydroxy-2-naphthoic acid, before undergoing dioxygenation and side chain removal to form chromone which was later converted into benzoic acid and CO_2_. This pathway contrasts with the common dioxygenation route observed in the free Laccase system, which is observed in the second degradation pathways. Furthermore, toxicity tests using *V. parahaemolyticus* and HT-22 cells, respectively, demonstrated the non-toxic nature of Laccase-ABTS-mediated metabolites. Intriguingly, analysis of the expression level of Alzheimer’s related genes in HT-22 cells exposed to degradation products revealed no induction of neurotoxicity unlike untreated cells. These findings propose a paradigm shift for bioremediation by highlighting the Laccase-ABTS system as a promising green technology due to its efficiency with the discovery of a potentially less harmful degradation pathway, and the production of non-toxic metabolites.

**Supplementary Information:**

The online version contains supplementary material available at 10.1007/s10532-024-10084-3.

## Introduction

﻿Polycyclic aromatic hydrocarbons (PAHs) are a ubiquitous class of persistent organo-pollutants consisting of two or more fused aromatic rings with a varying degree of carcinogenicity, genotoxicity, and mutagenicity (Slámová et al. 2017). They are formed during incomplete combustion, pyrolysis processes and from anthropogenic sources such as industrial food processing methods (heating, drying and smoking processes), home food preparation (grilling and roasting processes), municipal seep and oils spillages (Singh et al. 2016). Anthracene is considered a marker in the environmental assessment of PAHs, owing to its frequent detection in indoor suspended particles (air), surface water and groundwater that receive wastewater effluent (Adeniji et al. 2018). Primary target for anthracene includes the skin, hematopoietic system, lymphoid system, and gastrointestinal tract with varying degrees of toxicity and carcinogenicity (Hu et al. 2009). Also, its metabolites (quinones) have been implicated in acute and chronic toxicity of aquatic organisms, such as reduction in photosynthesis rate and population growth repression (Othman et al. 2012; Šepič et al. 2003).

Despite efforts from regulatory agencies like the United States Environmental Protection Agency, the European Union Scientific Committee on Food, the Joint FAO/WHO Expert Committee on Food Additives (JECFA) and the International Programme on Chemical Safety (IPCS), PAHs persist in the environment, necessitating new strategies to mitigate their acute and chronic effects (Zelinkova and Wenzl 2015). Several treatment methods, including physical, chemical, and biological methods or their combinations have been developed (Forján et al. 2020; Haneef et al. 2020). Among these, biological methods which relies on the use of microorganisms for enzymatic breakdown of PAHs offer many advantages such as low-cost, safety, reduced energy requirements, operation under milder conditions, and avoid generation of toxic compounds (Asemoloye et al. 2020). However, these methods have drawbacks, such as the need for long incubation times, mycelia ageing in the case of fungi, generation of a waste stream and possible formation of more toxic compounds (Ali et al. 2017).

To overcome these limitations, purified ligninolytic enzymes (Laccase, Manganese peroxidase and Lignin peroxidase) have been proposed as alternatives to whole cells owing to their low energy input requirement, moderate operational conditions, the specificity in the minimization of the undesirable products, ability to retain extremophilic property and stability under harsh conditions (Kumar and Chandra 2020).

Laccase ﻿(EC 1.10.3.2, benzenediol: dioxygen oxidoreductase) belonging to a multicopper oxidase family﻿ has become an attractive candidate for an eco-friendly degradation of persistent organo-pollutants into an innocuous state (Janusz et al. 2020) due to its stability, low cost, feasible production, broad substrate range and low substrate specificity (Agrawal et al. 2018). Compared to commercially available Laccases, isolated enzymes from whole cells offer several potential advantages. They are eco-friendly, utilizing molecular oxygen and yielding water as the sole by-product (Mayolo-Deloisa et al. 2020). Furthermore, the immobilization of Laccase enhances its stability and reusability, making it a more efficient and cost-effective option (Brandi et al. 2006; Fernández-Fernández et al. 2013; Ren et al. 2020). These factors, combined with its ability to oxidize a wide range of substrates, make isolated Laccase a superior choice for various industrial and environmental applications. However, recent studies highlight a critical challenge of the low degradation efficiency of Laccases towards PAHs due to their specific molecular structure (Xu et al. 2020). Redox mediators like 2,2′-azino-bis(3-ethyl-benzothiazoline-6-sulfonic acid) (ABTS) have shown promise in improving this degradation efficiency (Grassi et al. 2011; Wu et al. 2008).

According to available literature, a crucial gap in our understanding lies in the impact of these Laccase-mediated degradation products on living organisms. This lack of knowledge hinders a complete picture of the environmental safety of Laccase-based bioremediation. This study addresses this critical gap by investigating the oxidation and detoxification of anthracene by purified Laccases from two specific fungal strains: *Trichoderma lixii* FLU1 and *Talaromyces pinophilus* FLU12. We explore the significance of the Laccase-mediator ABTS system in enhancing anthracene degradation and elucidate degradation kinetics and pathways through metabolite profiling. Furthermore, to assess the environmental safety of this approach, we evaluate the acute toxicity of the resulting degradation products on a marine bacterium, *Vibrio parahaemolyticus*, which represents a potential ecological recipient of PAH contamination. Additionally, we investigate the cytotoxicity of these products on a mouse hippocampal neuronal cell line (HT-22) to explore potential neurotoxic effects, linking back to the established connection between PAHs and neurodegenerative diseases (Tang et al. 2003). Also, we analyze the effects on genes associated with Alzheimer's disease, providing further insight into potential neurotoxicity. By investigating these aspects, this study aims to comprehensively evaluate the environmental safety of Laccase-mediated PAH degradation, providing valuable insights for its potential application in bioremediation strategies.

## Material and methods

### Reagents

ABTS (2,2-azino-bis-(3-ethyl-benzothiazoline-6-sulphonic acid)), anthracene and ethyl acetate of analytical grade with ≥ 98% purity were purchased from Merck (St. Louis, MI, USA). The mouse hippocampal neuronal cell line was a gift from Salk Institute for Biological Studies, La Jolla, CA, USA. The PAH anthracene was selected for this study based on its toxicological profile as a known or reasonably anticipated human carcinogen on the US EPA’s priority pollutants list (Zelinkova and Wenzl 2015).

### Enzyme production and purification

Laccase production was carried out in cotton-plugged Erlenmeyer flasks (250 mL) containing basal salt medium (BSM) supplemented with anthracene (200 mg/L) as an inducer to a final volume of 150 mL prior to the set-up. The media were sterilized by autoclaving at 121 °C for 15 min. Inoculation was done directly into individual Erlenmeyer flasks using two 20 mm mycelial disks of each strain before incubating at 30 ℃ and shaking at 180 rpm for 10 days in complete darkness (MRC laboratory instrument, Essex, U.K). The pH of the flask containing strain *Tl*FLU1 was adjusted to 4 using 1 M HCl while that of *Tp*FLU12 was adjusted to 7 using 1 M NaOH. The rationale behind the variation in the culture media pH values was due to its optimized condition during anthracene degradation with 100% degradation at 12 days during our previous study. Also, the fungal strains whole cell showed tolerance to fluoranthene (Egbewale et al. 2023) and anthracene (data not shown) with zero growth inhibition at 400 mg/L of fluoranthene and 400 mg/L of anthracene for *Tl*FLU1 except for *Tp*FLU12 with no growth inhibition at 600 mg/L of anthracene. The detailed composition of the BSM media, including specific components and their concentrations, is provided in Table S1.

The purification was done according to the previously described method (Othman et al. 2018) with some modifications. Briefly, crude extract from *Tl*FLU1 (*Tl*FLU1L abbreviated for Laccase from *Tl*FLU1) and *Tp*FLU12 (*Tp*FLU12L abbreviated for Laccase from *Tp*FLU12) was individually centrifuged at 5000×*g* for 20 min at 4 ℃ to obtain a clear supernatant. Protein from each supernatant was sequentially precipitated to saturation with 60% (NH_4_)_2_SO_4_ under a gentle continuous stirring at 4 ℃ overnight. The protein pellets were recovered by centrifugation at 10,000×*g* for 20 min, dissolved in sodium acetate buffer (50 mM, pH 5) and dialyzed against a large volume of the same buffer using 30 kDa cut off size dialysis tubing cellulose membrane (Merck, Burlington, MA, USA). The protein was further purified using DEAE liquid chromatography column (1.5 × 9 cm) at room temperature before eluting with 100 mM NaCl in 50 mM sodium acetate buffer (pH 7) at a flow rate of 0.5 mL/min. Six fractions showing Laccase enzymes activity of substrate ABTS were pooled and applied on Sephadex G-100 column (2.0 × 9 cm) size exclusion column before elution with 50 mM sodium acetate buffer (pH 7). All fractions with Laccase activity were pooled, desalted, filter-sterilized, and stored at 4℃ until further usage.

The Laccase purity and molecular mass was visualized with SDS-PAGE using a 12% resolving gel and a 5% stacking gel. At the end of electrophoresis, the gel was stained with Coomassie brilliant blue (Laemmli 1970). The Laccases were purified to homogeneity with a specific activity of 34 U/mg protein. *Tl*FLU1L was characterized as a yellow Laccase while *Tp*FLU12L was a blue Laccase with a molecular mass of 44 kDa and 68.7 kDa, respectively. Both showing remarkable stability in various organic solvents, detergents, inhibitors, and metal ions with excellent residual activity.

**Fig. 1:**
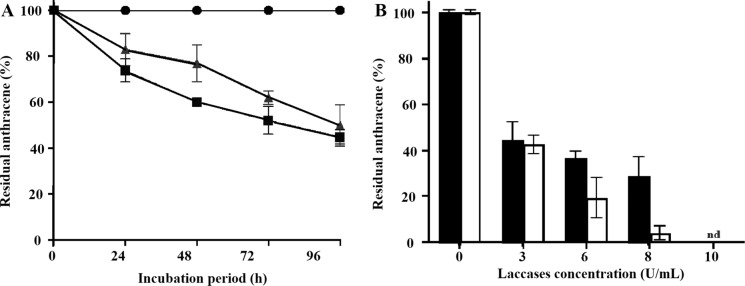
**A** The percentage of residual anthracene concentration after the oxidation by *Tl*FLU1L (filled square) and *Tp*FLU12L (filled triangle) and no enzyme controls (filled circle); **B** Effect of *Tl*FLU1L (filled square) and *Tp*FLU12L (open square) concentrations on oxidation of anthracene (200 mg/L), at pH 5, temperature 30 °C, after 96 h incubation period

### Assay for laccase activity

Laccase activity was measured as described previously (Zungu et al. 2020). Briefly, Laccase (370 μg) was mixed with 800 μL of 20 mM Na-Acetate buffer (pH 5) and 100 μL of 5 mM ABTS. After 15 min incubation period at 30 °C, the reaction was terminated with 50 μL of 20% (v/v) trichloroacetic acid (TCA) solution. One unit of Laccase is the amount of enzyme that produces 1 µM of oxidized product per minute (ε_420nm_ = 36,000 /M/cm for oxidized product).

### Degradation of anthracene by Laccases

Anthracene degradation was determined as previously described (Cambria et al. 2008) with some modifications. The reaction mixture (10 mL) consisted of anthracene (200 mg/L) and Laccase (2 U/mL, pH 5) in Na–Acetate buffer (50 mM, pH 5). Tween 80 (1%) was added to the mixture to enhance anthracene bioavailability and incubated at 30 °C in the dark for 96 h, with continuous shaking at 80 rpm to avoid precipitation. The pH of 5 was adopted for this study due to the observed 100% residual activity after 24 h reaction during the enzyme characterization assays in our previous study. The sample aliquots (1 mL) were drawn every 24 h and 50 μL of a 20% (v/v) TCA solution added to stop further enzymatic oxidation. All the experiments were performed in triplicates and a control (heat-denatured Laccase) set-up was carried out under the same experimental condition. The concentration of residual anthracene in the reaction mixture was measured as described previously (Mot et al. 2020) with slight modifications. Briefly, the reaction mixture aliquots were mixed with ethyl-acetate (1:4 v/v) in a separating funnel, vigorously shaken for 5 min and allowed to stand for 20 min to enhance the separation of aqueous and organic phases. The organic phase was collected, dried over 10 g anhydrous Na_2_SO_4_ and evaporated to dryness at 40 °C under reduced pressure. The dried fractions were then re-dissolved with the same extraction solvent and diluted tenfold with ethyl-acetate before quantifying the anthracene by measuring optical density at scanning mode from wavelength 200–400 nm as well as at wavelength 265 nm using Agilent Cary60 UV–Vis spectrophotometer (Santa Clara, CA, USA).

### Detection of the metabolites by GC–MS analysis

The extracted fractions from the aliquots collected were pooled together, dried at 40 °C under reduced pressure and redissolved to a final volume of 1 mL with *n-*hexane. 1 µL of samples were injected into Rxi-PAH capillary GC column (GCMS-QP2010 SE Shimadzu scientific, Kyoto, Japan) for metabolite detection under the following conditions: split injection (injector temperature 330 °C, split 1/8 for samples and 1/20 for standard samples); oven temperature programmed from 60 °C (held for 2 min) to 300 °C (15 min) at 10 °C/min. The carrier gas used was helium at a flow rate of 1 mL/min. The metabolites were analyzed at a full scanning mode at 10–220 m/z using the NIST library (National Institute of standard technology, USA) (Teh and Hadibarata 2014). ﻿

### Influence of Laccase-mediator system on anthracene degradation

The influence of ABTS as a mediator on anthracene oxidation was determined by adding ABTS at a varying concentration (200 µM to 10 mM) to the Laccase solution (2 U/mL). Subsequently, anthracene (200 mg/L) was correspondingly added into the Laccase-mediator solutions in a final volume of 10 mL containing Na-Acetate buffer (50 mM) and Tween 80 (1%). Each reaction mixture solution was adjusted to pH 5 and incubated in the dark at 30 °C, with shaking at 80 rpm for 96 h. Aliquots (1 mL) were drawn from each reaction every 24 h and residual anthracene concentration, metabolite detection and change in the functional group of the detected metabolite were determined as described above. All the experiments were performed in triplicates and a control (heat denatured enzyme) set-up was carried out under the same experimental condition. It is worth noting that the choice for ABTS as a mediator, concentration (200 µM to 10 mM and pH 5 condition were based on the higher binding activity of the Laccases towards ABTS in comparison to other mediators during the enzyme characterization study, which aligns with previous studies Camarero et al. 2008; Liu et al. 2017), where optimum pH for oxidation of ABTS was reported to range between 4 and 7 with the highest activity with ABTS. The stability and reusability of ABTS@MIL-100(Fe) as a Laccase mediator make it a promising tool for dye removal (Liu et al. 2017).

### Determination of kinetic parameters of the Laccase-mediator system

The kinetic parameters of the Laccase and Laccase-mediator were determined using anthracene concentrations ranging from 50 to 400 mg/L, following the assays described above (de Freitas et al. 2017). A non-linear regression model was used to estimate the Michaelis–Menten constant *K*_m_ and *v*_max_ for the substrate disappearance. The changes in the anthracene concentrations were monitored over a 96-h period immediately after reaction initiation by the addition of 3 U Laccase and 10 mM ABTs (for Laccase-mediator set-up) at 30 °C. Anthracene concentrations above 400 mg/L could not be used due to the solubility issue.

### Ecotoxicity analysis

Ecotoxicity analysis of the anthracene degradation products (metabolites) was carried out to assess the potential risk on biota using time-course acute toxicity effects on a marine bacterium, *Vibrio parahaemolyticus* (ATCC 17802) as a model organism, being the primary consumer in the food web. The acute toxicity of pure anthracene (control), Laccase degraded and Laccase-mediator (degradation products/h) on *V. parahaemolyticus* (100 μL of diluted standardized overnight culture broth) was monitored based on a modified liquid-to-plate micro-counting method as described previously (Egbewale et al. 2023; Rotini et al. 2018).

### Cytotoxicity assay

The cytotoxicity assay was performed using mouse hippocampal neuronal (HT-22) cell line as the secondary consumer. The time course cytotoxic effect of the degradation product on HT-22 cells was carried out using an MTT assay. The MTT assay was based on the colorimetric test of cell proliferation and percentage cell viability of the degradation product/h. Briefly, cells maintained in DMEM supplemented with 10% FBS and 100 mg/L streptomycin and 100 IU/mL penicillin at 37 °C in a humidified atmosphere of 5% CO_2_ were plated at a density of 1.28 × 10^5^ cells/mL in 96 well plates. The cells were exposed to each degradation product (1:4 v/v) of 400 mg/L anthracene concentration in a 96-well plate and allowed to incubate for 48 h. Metabolites from the degradation of anthracene at an initial concentration of 400 mg/L were used due to the presence of residual anthracene at the end of the reaction period.

### Cell morphology evaluation

The evaluation of HT-22 cells for viability, morphology and overall health was done by capturing multiple field views for each experimental condition to ensure representative analysis using a high-resolution microscope, Invitrogen EVOS™ Floid EN61326 equipped with phase contrast or fluorescence capabilities (Thermos-Fisher Scientific, Waltham, MA, USA).

### Alzheimer’s related gene expression analysis

Alzheimer’s related gene expression analysis in HT-22 cells incubated with control (pure anthracene), oxidation products of 72 h (anthracene 37 mg/L and 32 mg/L incubated with 3 U of *Tl*FLU1L and *Tp*FLU12L, respectively) and 96 h (anthracene 139 mg/L and 58 mg/L incubated with 3 U of *Tl*FLU1L and *Tp*FLU12L, respectively) was performed using RT-qPCR (CFX Connect Real-Time PCR System, Bio-Rad CFX 976, Hercules, CA, USA). Total RNA from HT-22 cells was extracted using GeneJET RNA purification Kit (Thermos-Fisher Scientific, Waltham, MA, USA). cDNA was synthesized from 100 ng of total RNA using the Maxima First-strand cDNA Synthesis Kit (Thermos-Fisher Scientific, Waltham, MA, USA). RT-qPCR conditions were as follows: 40 cycles of 95 °C for 30 s, 95 °C for 0.05 s, 60 °C for 30 s, and 72 °C for 20 s. Melting curve analysis was performed from 65 to 95 °C. The primer pairs used are shown in Table 1. Triplicate analyses were carried out for each cDNA sample along with the ‘no reverse transcriptase’ and ‘no template’ controls. The specificity of the amplicons was confirmed through melting curve analysis and size determination on 2% agarose gels. Gene expression was quantified using the relative standard curve method. Different dilutions of cDNA synthesized from RNA extracted from untreated HT-22 cells were used to plot the standard curves for each gene. BACE-1, ADAM-10, TAU, PPARγ and APP mRNA expression were presented as fold regulation (negative inverse of fold change).

**Table 1:** Primers sequences to amplify genes associated with Alzheimer's disease in HT-22 cells

Genes		Primers (5′–3′)	Size (bp)	References
Housekeeping gene β-actin (ACTB)	F	ATCTGGCACCACACCTTCTACAATGAGCTGCG	120	Hu et al. (2022)
	R	CGTCATACTCCTGCTTGCTGATCCACATCTGC		
ADAM-10 (a disintegrin and metalloproteinase)	F	GCCAGTTCTGATGGCAAAGATG	90	Ao et al. (2022)
	R	AGACCCTGTACTGCCACAAGTTGA		
TAU (microtubule-associated protein tau)	F	CAAGTGTGGCTCAAAGGACAATATC	55	Yi et al. (2019)
	R	TCAATCTTCTTATTCCCTCCTCCAG		
PPARγ (peroxisome proliferator-activator recepter gamma)	F	CTCCCAGCTGTCGCAAGGTGC	174	Pesant et al. (2006)
	R	GCAATCGATAGAAGGAACACT		
APP (amyloid precursor protein)	F	AATGTGGATTCTGCTGATGCGGAG-	657	Ko et al. (2004)
	R	CCCATTCTCTCATGACCTGGGA		
BACE-1 (beta-secretase 1)	F	CATTGGAGGTATCGACCACTCGCT	624	Tamagno et al. (2006)
	R	CCACAGTCTTCCATGTCCAAGGTG		

F = Forward, R = Reverse

### Quality control measures

#### Recovery study

We have included a recovery study using sterile Na-acetate buffer with heat-denatured Laccase spiked with a known concentration of anthracene as standard. This step assesses potential losses during sample preparation and extraction. The average recovery of anthracene in our study was 98.7 ± 3.53%.

#### Standard curve

A standard curve was generated using known anthracene concentrations (with an R^2^ value of 0.972) to ensure accurate quantification of residual anthracene in the reaction mixtures.

#### Triplicate experiments

All experiments were performed in triplicates to account for experimental variability and enhance data reliability.

#### Control experiment

A control experiment using heat-denatured Laccase was included under the same conditions as the treatment groups to account for non-enzymatic degradation of anthracene.

#### Blank control

Blank controls included a well containing all reaction components except the enzyme sample. This controls for background absorbance at the measurement wavelength.

#### Substrate controls

Substrate controls included wells with only substrate and buffer (no enzyme) and only enzyme and buffer (no substrate) to assess potential non-enzymatic conversion of the substrate and background enzyme activity, respectively.

#### Serial dilution of enzyme

A serial dilution of the enzyme sample was carried out to ensure the measured activity falls within the linear range of the assay.

#### Cell viability assay

Cell viability assay alongside the ecotoxicity test was conducted to distinguish between growth inhibition and cell death caused by the degradation products.

#### No template control (NTC)

NTV included a reaction mixture containing all components except the cDNA template. This controls for potential contamination from reagents or amplification of nonspecific products.

#### Positive control

A reference gene (β-actin) with known stable expression in the HT-22 cell line was used to verify the functionality of reverse transcription and PCR steps.

#### Melt curve analysis

Melt curve analysis after amplification was carried out to ensure the generation of a single specific product for each gene of interest.

#### Efficiency testing

The amplification efficiency of each primer was measured using a standard curve to ensure accurate quantification of gene expression levels.

#### Anthracene and ABTS treatments after studies

The anthracene and ABTS used in the experiment were treated according to the South African Department of Water and Forestry (DWAF 1996; Pillay and Olaniran 2016) waste treatment protocol and standards. After the reaction with Laccase, the anthracene and ABTS were neutralized and safely disposed of per environmental regulations to prevent potential environmental issues. Although the degradation products of ABTS were not found to pose a risk to the environment yet we took care of it to ensure that all waste generated during the experiment was properly managed to minimize any adverse impact on the environment.

### Statistics analysis

All measurements were carried out in triplicates. The data were expressed as the means ± standard deviations. Statistics was performed using the Graph-Pad Prism v3.0 (Boston, MA, USA). To determine the *K*_m_ and *v*_max_, experimental data was fitted to reciprocal form of the Michaelis–Menten equation (*v* = *v*_max_[S]/(*K*_m_ + [S])) using Graph-Pad Prism v3.0.

## Results

### Oxidation of anthracene by Laccases

The residual anthracene (%) after oxidation by purified Laccase from *Trichoderma lixii* FLU1 (*Tl*FLU1L) and *Talaromyces pinophilus* FLU12 (*Tp*FLU12L) is shown in Fig. 1A and Table S2. Both enzymes reduced the anthracene concentration during the 96-h incubation period. *Tl*FLU1L (2 U/mL) and *Tp*FLU12L (2 U/mL) could oxidise 55.1% and 49.9% of anthracene (200 mg/L) as 44.9% and 50.1% of anthracene concentration was recorded after 96 h incubation, respectively. As the concentration of Laccases increases, a significant reduction in the concentration of anthracene was observed after 96 h incubation period (Fig. 1B, Table S3). 3 U/mL of *Tl*FLU1L and *Tp*FLU12L could oxidise 55.7% and 57.5% while 8 U/mL of respective enzyme oxidized about 71.3% and 96.1% of anthracene, respectively, after 96 h incubation period. No residual anthracene was detected when 10 U/mL concentration of Laccases was used, indicating 100% anthracene oxidation.

### Oxidation of anthracene by Laccases in the presence of ABTS

**Fig. 2:**
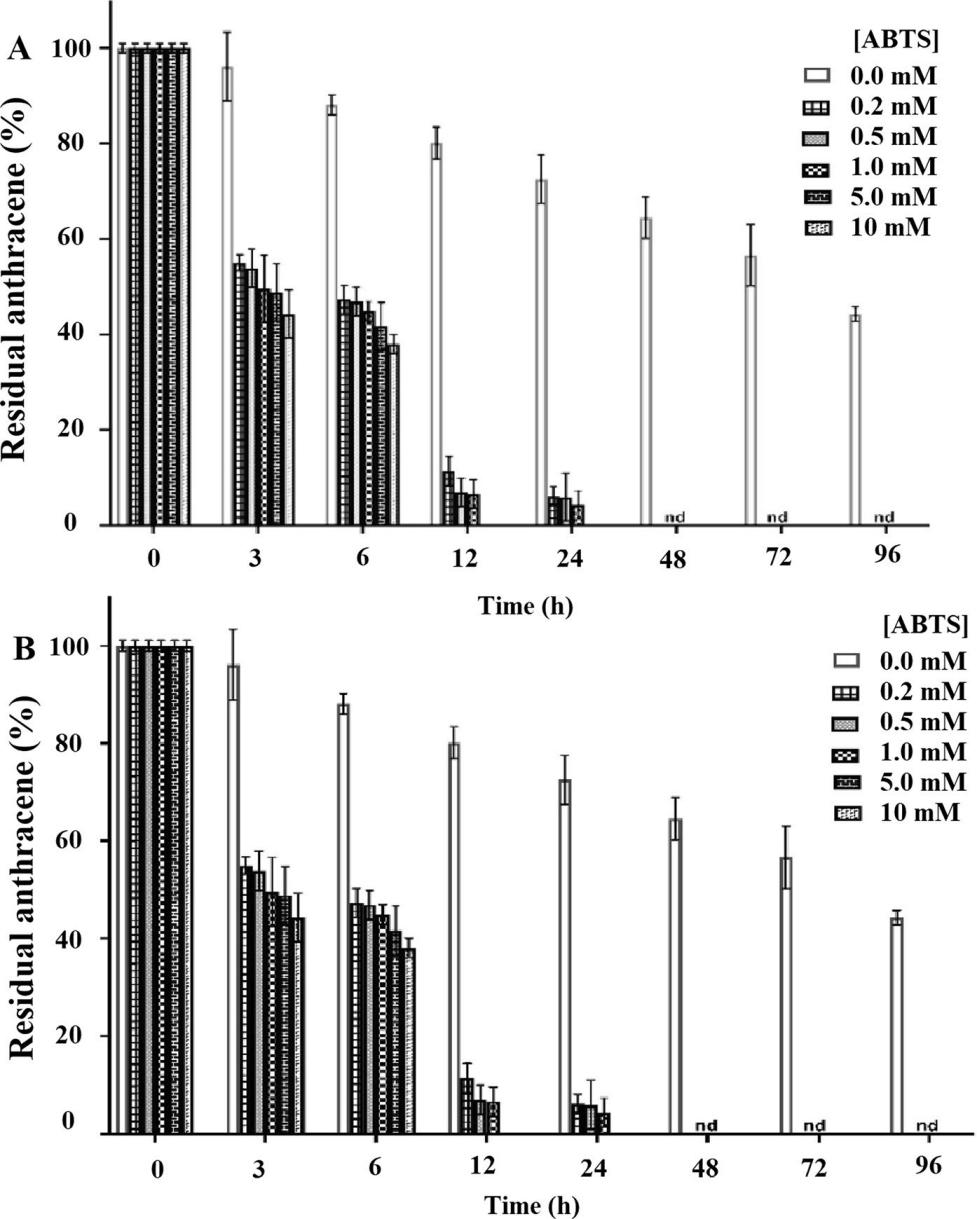
The influence of mediator (ABTS) concentration on *invitro* oxidation of anthracene by *Tl*FLU1L (**A**) and *Tp*FLU12L (**B**)

The in vitro oxidation of anthracene in the presence of varying concentration of mediator (ABTS) by purified *Tl*FLU1L and *Tp*FLU12L is shown in Fig. 2A, B. 2 U/mL of purified *Tl*FLU1L (Fig. 2A, Table S4) and *Tp*FLU12L (Fig. 2B, Table S5) incubated with ABTS concentration (10 mM) before adding to 200 mg/L of anthracene showed higher reduction in the anthracene as compared to when only enzymes was added to the reaction. Addition of 200 µM ABTS drastically increased the anthracene degradation by both enzymes just after 3–24 h of incubation period. There was no residual anthracene concentration observed after 6 h when 5–10 mM of ABTS were added to the reactions. *Tp*FLU12L could reduce anthracene concentrations to 4.1% and 6.0% after 24 h of incubation when 5 or 10 mM of ABTS was added, respectively. The results indicate that just after 3 h of incubation, 200 µM of ABTS enhances anthracene degradation by 41% and by 100% after 12 h in presence of 5–10 mM of ATBS in case of *Tl*FLU1L.

### The degradation kinetics analysis

**Fig. 3:**
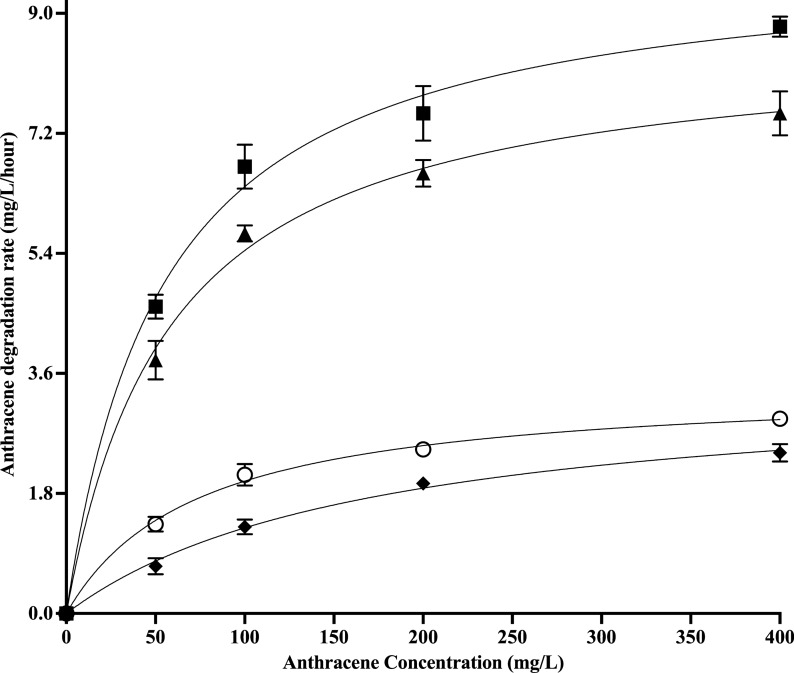
Laccase-ABTS kinetics of anthracene (200 mg/L) oxidation by *Tl*FLU1L (filled diamond), *Tp*FLU12L (open circle), *Tl*FLU1L + ABTS (filled triangle) and *Tp*FLU12L + ABTS (filled square). Conditions: pH 5, temperature 30 °C, Laccases concentration (3 U/mL), ABTS concentration (10 mM) and incubation period 96 h

The degradation kinetics of anthracene mediated by *Tl*FLU1L and *Tp*FLU12L in the absence and presence of mediator (ABTS) is presented in Fig. 3 and Table 2. In the absence of ABTS, *v*_max_ values of 3.51 ± 0.06 mg/L/h and 3.44 ± 0.06 mg/L/h with *K*_m_ 173.2 ± 0.06 mg/L and 73.3 ± 0.07 mg/L were recorded for *Tl*FLU1L and *Tp*FLU12L, respectively, during in vitro degradation of anthracene. Interestingly, in the presence of a mediator (ABTS), *v*_max_ values increased to 8.63 ± 0.16 mg/L/h and 9.89 ± 0.22 mg/L/h while *K*_m_ values decreased to 58.5 ± 0.19 mg/L and 54.6 ± 0.25 mg/L, respectively, for *Tl*FLU1L and *Tp*FLU12L (Fig. 3, Table 2).

**Table 2:** Michaelis–Menten kinetic parameters of the in vitro degradation of anthracene by *Tl*FLU1L and *Tp*FLU12L

	*v*_max_ (mg/L/h)		*K*_m_ (mg/L)		*R*^2^	
	*Tl*FLU1	*Tp*FLU12	*Tl*FLU1	*Tp*FLU12	*Tl*FLU1	*Tp*FLU12
Anthra	3.51 ± 0.06	3.44 ± 0.06	173.2 ± 0.06	73.3 ± 0.07	0.997	0.997
Anthra + ABTS	8.63 ± 0.16	9.89 ± 0.22	58.5 ± 0.19	54.6 ± 0.25	0.997	0.996

Value in each column is the mean ± standard error. Anthra: Anthracene

### The proposed degradation pathway depicted by GC–MS

**Fig. 4:**
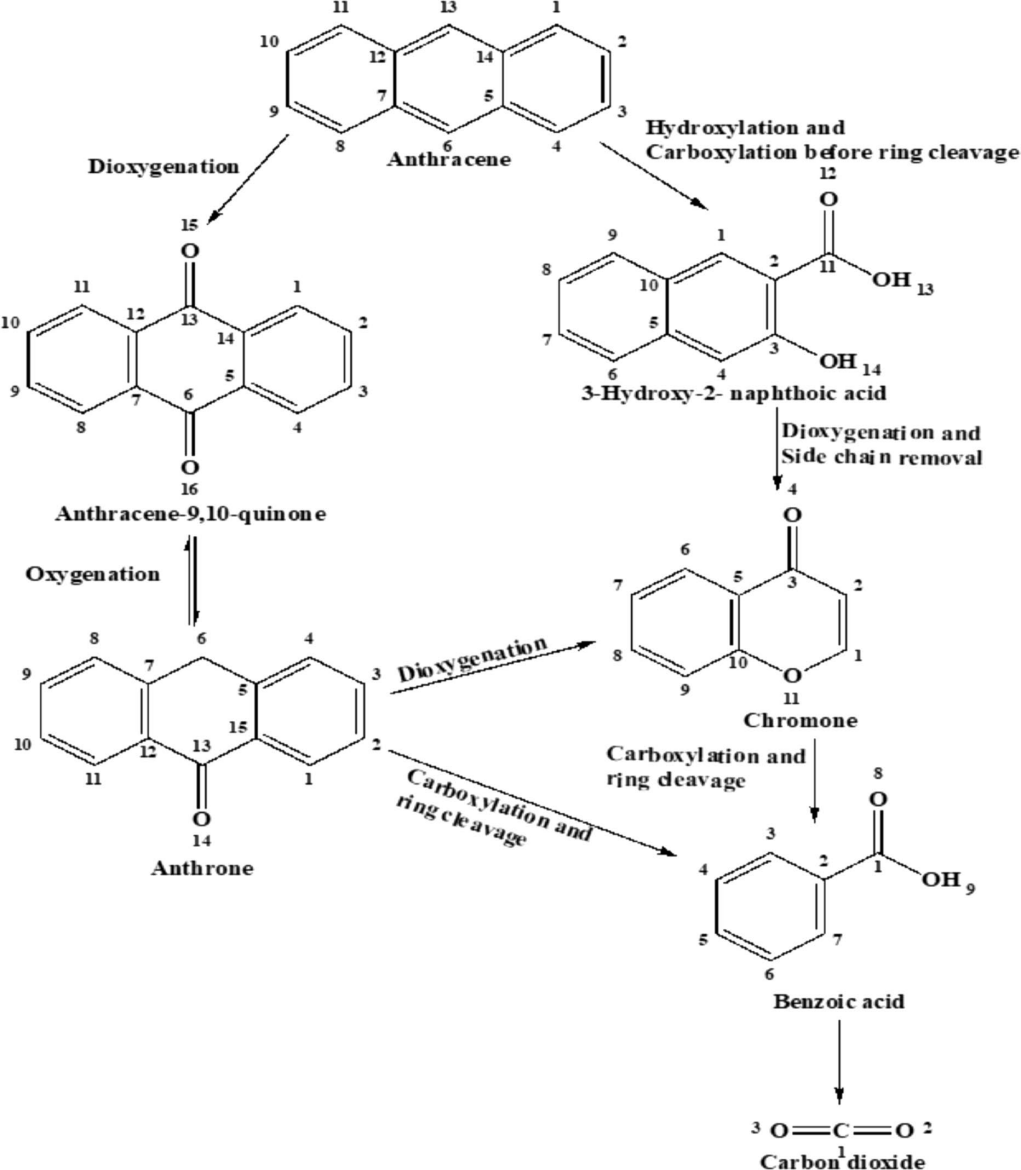
A proposed pathway for anthracene degradation by *Tl*FLU1L and *Tp*FLU12L

The GC–MS data analysis proposed that during anthracene degradation, five metabolites were detected which are anthracene-9,10-quinone at a retention time (RT): 16.1 min and m/z: 180) and benzoic acid (RT: 9.3 min and m/z: 105), respectively, in *Tl*FLU1L incubated tube and 3-hydroxy-2- naphthoic acid (RT: 17.5 min and m/z: 170), anthrone (RT: 11.8 min and m/z: 194), chromone (RT: 11.1 min and m/z: 146), benzoic acid (RT: 9.3 min and m/z: 9.3) in *Tp*FLU12L incubated tube. Based on the theoretical arrangement of the detected transient products, anthracene degradation was proposed to undergo two metabolic pathways, namely hydroxylation and carboxylation of C-1 and C-2 position of anthracene to form 3-hydroxy-2-naphthoic acid, before undergoing dioxygenation and side chain removal to form chromone which was later converted into Benzoic acid and CO_2_ release as a dead-end product. In addition, the second route was only observed in the absence of the mediator (ABTS) and proceeded via dioxygenation at C9–C10 position to form anthracene-9,10-quinone before its further conversion to anthrone via oxygenation which is broken down into chromone through dioxygenation and undergo carboxylation to form benzoic acid as a dead-end product (Fig. 4)*.*

### Ecotoxicity and cytotoxicity analysis of the metabolites of anthracene degradation

**Fig. 5:**
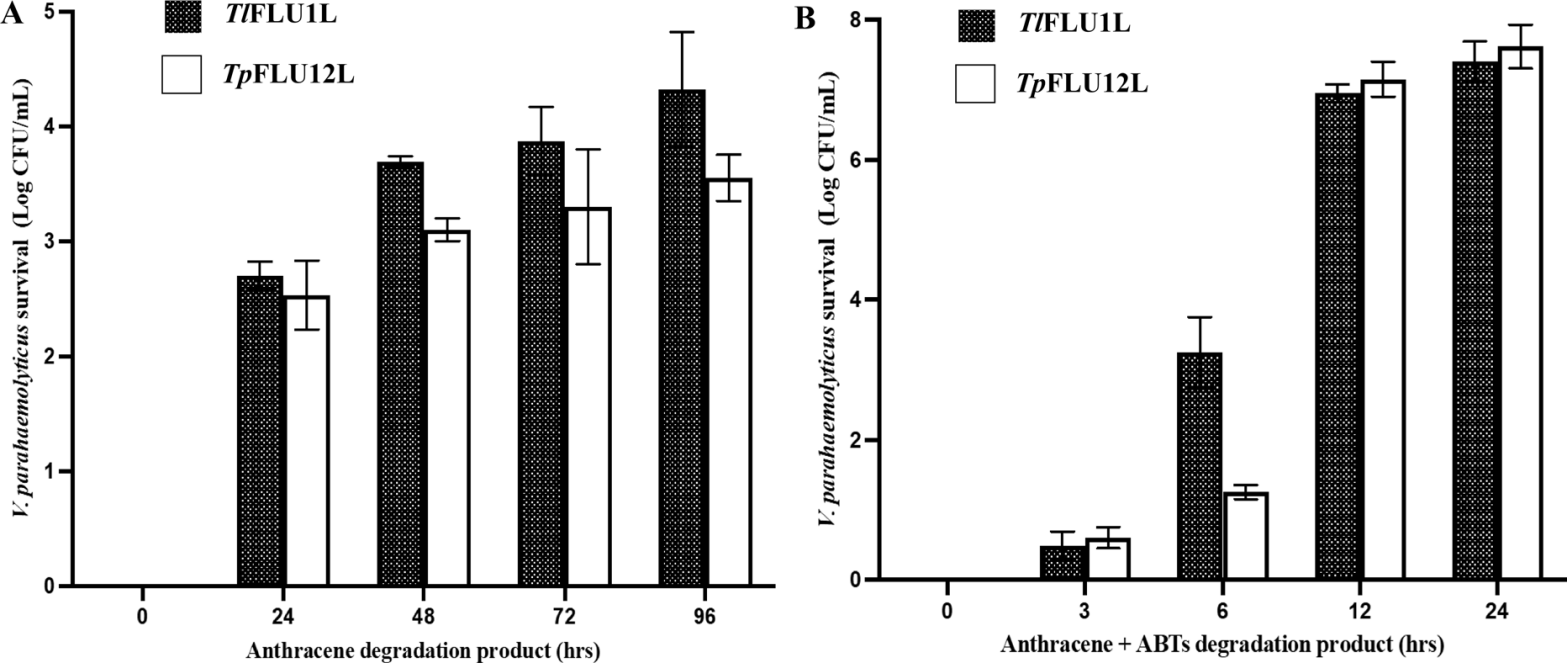
Time-course changes in toxicity of anthracene degradation products of *Tl*FLU1L, *Tp*FLU12L, *Tl*FLU1L + ABTS and *Tp*FLU12L + ABTS on *V. parahaemolyticus* (log CFU/mL)

Time-course changes in toxicity of anthracene degradation metabolites on *V. parahaemolyticus* (log CFU/mL) are presented in Fig. 5A, B. In the absence of a mediator (ABTS), the marine bacterium survival was recorded at 53.8% and 44.3%, in assays containing *Tl*FLU1L and *Tp*FLU12L, respectively, and the percentage survival increased with an increase in the number of days when the degradation products were collected. However, a significantly high survival of *V. parahaemolyticus* (7.9 log CFU/mL) was obtained with metabolites collected from the degradation assay in presence mediator (ABTS) after 24 h of incubation.

**Fig. 6:**
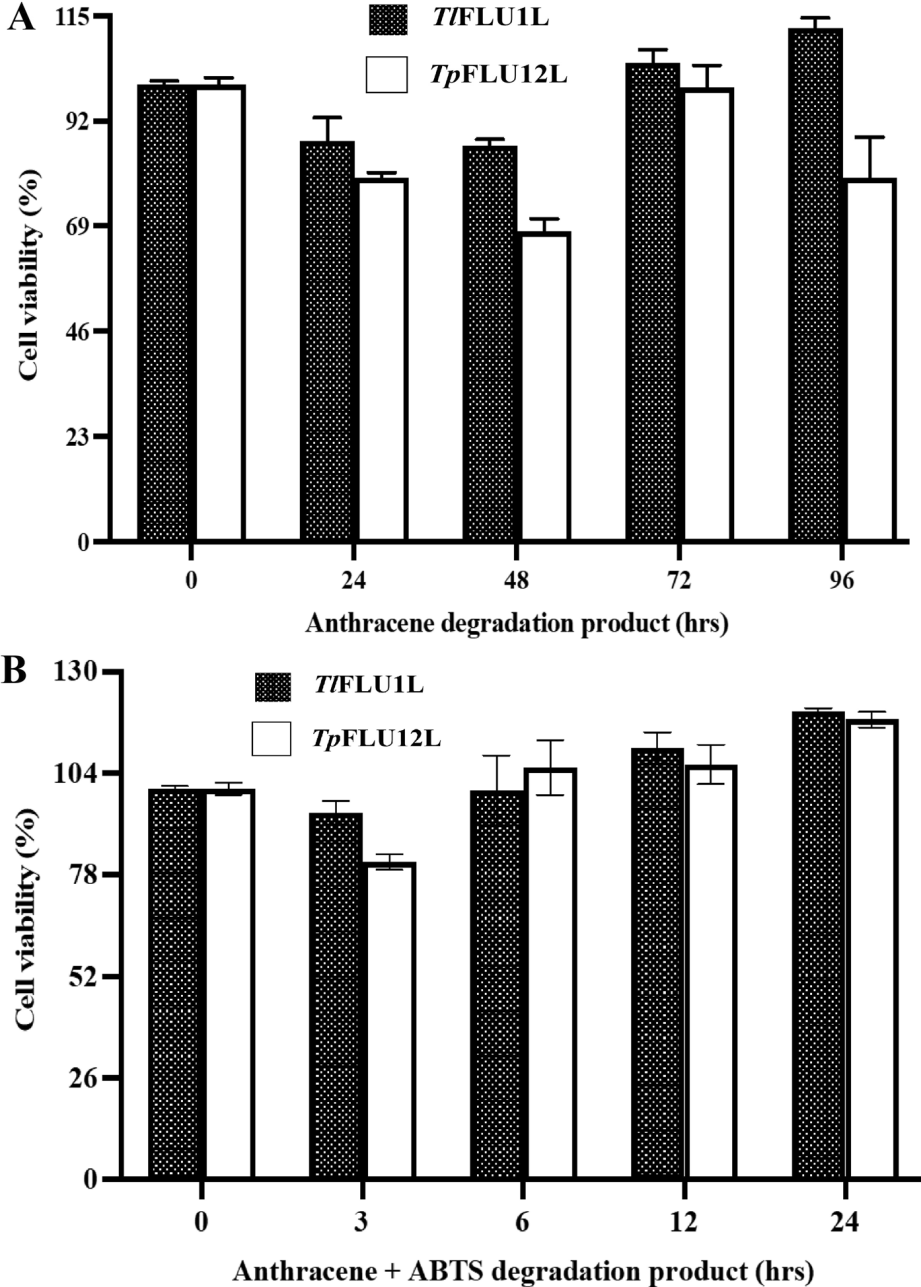
Time-course changes in toxicity of anthracene degradation products of *Tl*FLU1L, *Tp*FLU12L, *Tl*FLU1L + ABTS and *Tp*FLU12L + ABTS on on HT-22 cell-line

The time-course changes in the cytotoxic effect of anthracene degradation metabolites on HT-22 cells are presented in Fig. 6A, B. Without the mediator ABTS, a slight reduction in the cell viability (10% and 13% for metabolites collected after 24 h and 48 h, respectively) during anthracene degradation by *Tl*FLU1L, while metabolite from *Tp*FLU12L degradation resulted in 86% cell viability (for 24 h degradation product) and 70% cell viability (for 48 h degradation product). However, cell viability of 100% and 93% were obtained after 72-h degradation with *Tl*FLU1L and *Tp*FLU12L, respectively. In the presence of the ABTS mediator, up to 115% and 106% cell viability was obtained when treated with metabolites obtained after 24 h degradation by *Tl*FLU1L and *Tp*FLU12L, respectively.

**Fig. 7:**
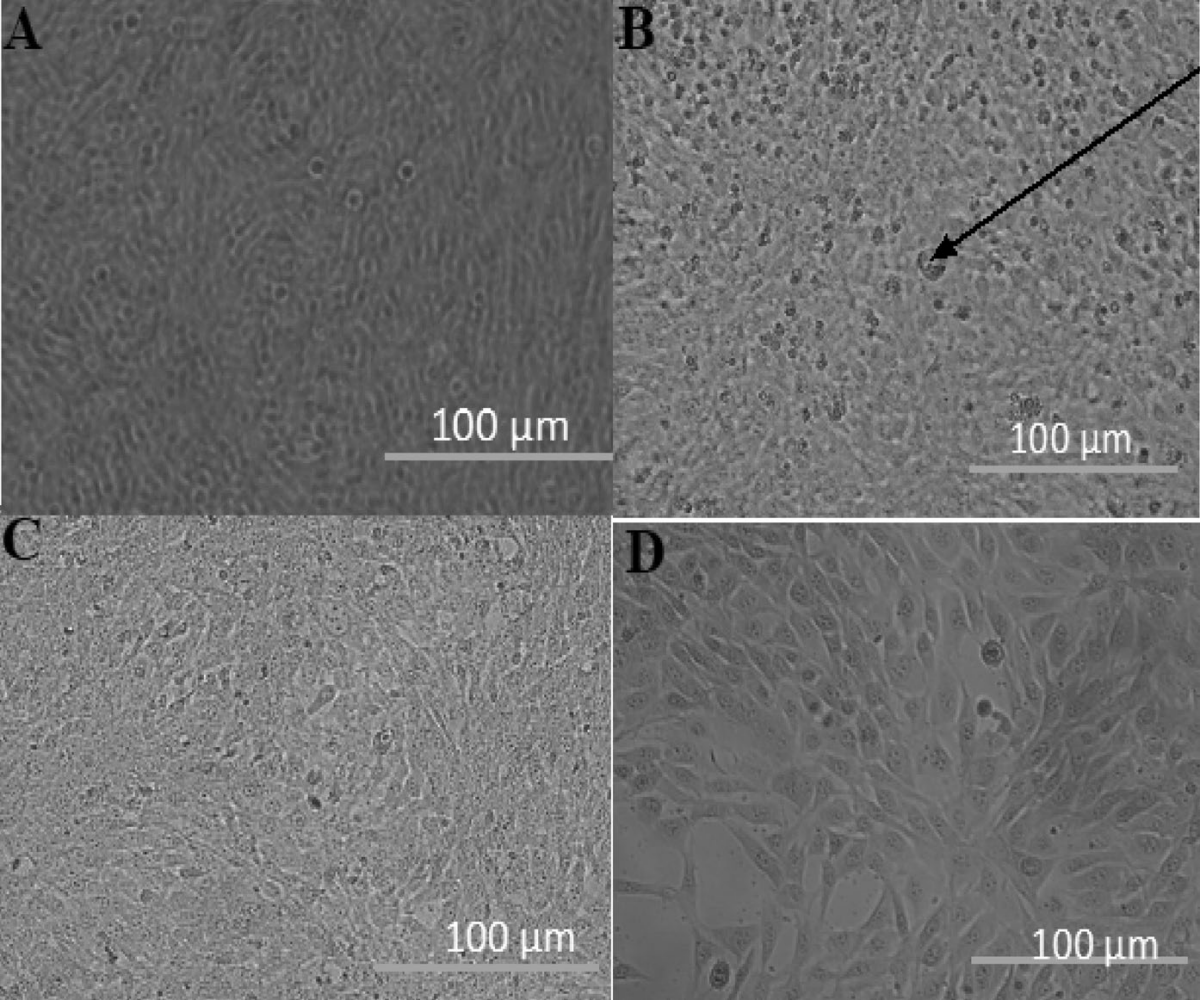
The micrograph of the HT-22 cell-line grown on anthracene degradation products. **A** Control (Blank), **B** Control (Anthracene), **C** *Tl*FLU1L treated cell line and **D** *Tp*FLU12L treated cell line. Arrow shows dead cells

Micrograph analysis of HT-22 cells treated with fluoranthene oxidation product from *Tl*FLU1L and *Tp*FLU12L is shown Fig. 7A, B. A distinct difference was noted between the control (blank) and HT-22 cells treated with anthracene oxidation product. In the control group and HT-22 cells treated with anthracene + ABTS oxidation product from *Tl*FLU1L and *Tp*FLU12L, cells exhibited a healthy morphology with a characteristic neuronal-like appearance with elongated, spindle-shaped bodies, well-defined nuclei, and uniform neurite processes. In contrast, the treated cells with anthracene only (positive control) and anthracene oxidation product from *Tl*FLU1L and *Tp*FLU12L, exhibited cytotoxic effects with cellular shrinkage, increased membrane blebbing, and loss of neurite extensions. The cells appeared smaller, with disrupted cell membranes and fragmented neurites, indicating impaired connectivity and reduced cell viability. Overall, the treated cells displayed decreased density, suggesting cell death caused by oxidation products.

**Fig. 8:**
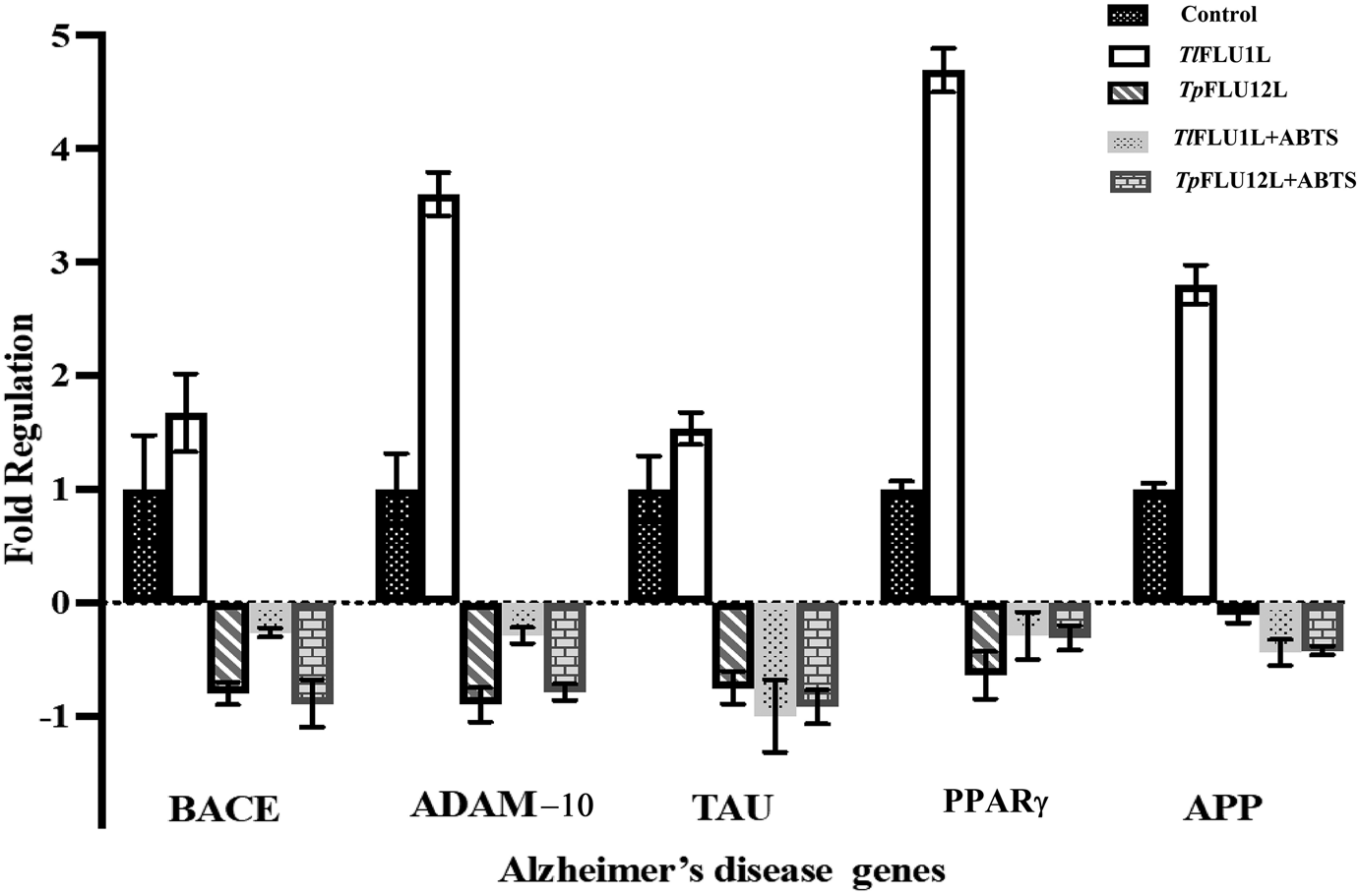
Effect of anthracene degradation products on expression of genes linked with Alzheimer’s in HT-22 cells

The effect of anthracene degradation metabolites on genes associated with Alzheimer's disease in HT-22 cells is presented in Fig. 8. The expression of genes BACE-1 (1.7-fold), ADAM-10 (3.6-fold), TAU (1.5-fold), PPARγ (4.7-fold) and APP (2.8-fold) genes were upregulated in cells treated with degradation products of *Tl*FLU1L 1 in comparison with the cell treated with degradation product of control group (Uncatalyzed anthracene). Conversely, in the degradation products of *Tp*FLU12L, *Tl*FLU1L + ABTS and *Tp*FLU12L + ABTS, down-regulation of BACE-1, ADAM-10, TAU, PPARγ and APP was observed.

## Discussion

The results of in-vitro enzymatic degradation of anthracene lend credence to the report that purified Laccase has broad substrate specificity due to its ability to degrade a wide range of aromatic compounds like PAHs (Ezike et al. 2021; Wu et al. 2010). The obtained low residual anthracene concentration in the tube incubated with purified Laccase from *Tl*FLU1 (*Tl*FLU1L) compared to that of purified laccase from *Tp*FLU12 (*Tp*FLU12L) can be linked to the intrinsic properties of the yellow Laccase from *Tl*FLU1 (*Tl*FLU1L) such as its structure/specific amino acid composition which could render it to be more efficient in catalyzing the oxidation of anthracene's aromatic rings than their blue counterpart. Pozdniakova et al. (2006) opined that the yellow Laccase is superior in degradation efficiency compare to its blue counterparts leading to into an innocuous state in the absence of mediators. Likewise, the low residual anthracene concentration in the tube incubated with *Tl*FLU1L could be attributed to its enigmatic preference for radical-forming aromatic substrate like PAHs which in return ﻿boosts its enzyme activity and stability during the catalytic reaction (Fig. 1A) (Mot et al. 2020).

Laccase concentration played a significant role in the degradation process as evident by the observed low residual anthracene concentration with an increase in *Tl*FLU1L concentrations (Fig. 1B) aligns with the expected enzyme–substrate kinetics. Higher Laccase concentration provides more catalytic sites available for anthracene oxidation, leading to faster degradation rates. This observation is consistent with previous studies on Laccase-mediated PAH degradation where efficient degradation of several PAHs increase with an increase in pure Laccase concentration during an aged soil amendment (Wu et al. 2008). Some authors have demonstrated that PAHs degradation in water is proportional to Laccase concentration in the beads of a Laccase-loading spider-type reactor (LSTR) (Niu et al. 2013). Also, the removal of PAHs is known to be influenced by low ionization potentials (IPs) (Kadri et al. 2017) while anthracene having an IP of 7.43 eV (Kukhta et al. 2009) could explain the rapid degradation of anthracene. Furthermore, PAHs with IP ≤ 7.55 eV are more susceptible to enzymatic degradation by fungi to quinonic metabolites, a substrate easily mineralized to CO_2_ by microbial populations and could naturally undergo polymerization to become part of the humus pool (Pozdnyakova 2012). The mechanism for PAHs degradation by Laccase could be attributed to Ips and Laccase can effectively degrade PAHs with IP below 7.45 eV (Li et al. 2010b).

Although several purified Laccase from fungi have been reported to degrade PAHs, its high production cost is a major challenge for commercial application. Hence, it becomes imperative to assess the influence of mediators on low Laccase concentration as an alternative to reduce high Laccase concentration usage. The observed low residual anthracene concentrations at 3 U of *Tl*FLU1L and *Tp*FLU12L in the presence of high mediator (ABTs) concentration suggests a synergistic effect between Laccase and mediator leading to a rapid generation of ABTS radicals due to high Laccase activity which then promote an attack at the PAHs benzene rings. This observation is consistent with the report of Naghdi et al. (2018) where ABTS was demonstrated to acts as a redox mediator by readily accepting electrons from laccase and transferring them to xenobiotics, facilitating its oxidation and subsequent degradation. Another possible explanation for this observation is that ABTS could function as an “electron shuttle” between the free enzyme and the substrate. This aligns with the report of (Dodor et al. 2004), where the mechanism of overall catalytic efficiency of Laccase in degrading anthracene was linked to ABTS acting as an “electron shuttle” between Laccase and the substrate (anthracene).

The degradation kinetics reaction of anthracene and *Tl*FLU1L under free- and mediator system revealed that the reaction undergoes first-order kinetics, where the rate of the reaction was independent of the substrate concentration but dependent on the amount of the enzyme used. This observation is in accordance with a previous report (Dodor et al. 2020) where PAHs degradation by fungi Laccase obeys first-order kinetics. The relatively lower *K*_m_ values of the mediator immobilized Laccases compared to the free enzyme indicate a potential conformational change of the enzyme due to mediator immobilization, leading to a higher affinity for ABTS used as a substrate for the enzyme activity assay. Likewise, the oxidization state(s) of ABTS and the pH of the reaction medium (acidic) can be considered as major factors which influenced the anthracene degradation (Li et al. 2010a).

Interestingly, two possible pathways were proposed for anthracene degradation by the Laccases investigated in this study. The first degradation route is initiated in the presence of the mediator alone (Table 3) with hydroxylation and carboxylation with ring cleavage at the C-1 and C-2 position of anthracene to form 3-hydroxy-2-naphthoic acid, before undergoing decarboxylation and side chain removal to form chromone which is later converted to benzoic acid and carbon dioxide through carboxylation and ring cleavage as a dead-end product. This same degradation product in our proposed pathway has been documented during anthracene metabolism in a halophilic bacterium, *Martelella* sp. AD-3, suggesting that it is a common metabolic pathway for anthracene (Cui et al. 2014). Conversely, the second degradation pathway was mainly observed during the free Laccase degradation of anthracene which proceeded through dioxygenation of C-9, and the C-10 position of the anthracene ring to form anthracene-9,10-quinone which is later converted to anthrone via oxygenation before yielding benzoic acid as a dead-end degradation product. This same degradation pathway has been identified during anthracene degradation by marine fungi *Cladosporium sp*. CBMAI 1237 (Birolli et al. 2018). Also, the appearance of only anthracene-9,10-quinone in the *Tl*FLU1L tube and anthrone alone in the *Tp*FLU12L tube suggest that those products are not stable and agree with various researchers who have well-confirmed anthraquinone/anthrone as a dead-end metabolite of anthracene (Aranda et al. 2017). The observation in the *Tp*FLU12L tube is consistent with a previous report (Jove et al. 2016) where fungi ligninolytic enzymes initiate anthracene degradation at C-9 and C-10 positions to form anthrone, which was later converted to benzoic acid.

**Table 3:** GC–MS profile of the metabolites accumulated during anthracene degradation by *Tl*FLU1L and *Tp*FLU12L

﻿M	Retention	Major m/z of fragment ions	Tentative metabolite	Laccase source	
	Time (min)	(% relative abundance)	Identification	*Tl*FLU1L	*Tp*FLU12
1	19.8	76 (6, **M**^**+**^), 88 (4), 89 (8), 150 (4), 151 (6), 152 (7), 176 (14),177 (8),**178** (100), 179 (16)	Anthracene	xx	
2	17.5	63 (14, M^**+**^), 71 (25), 88 (12), 113 (18), 115 (16),	3-Hydroxy-2-naphthoic acid	x	xxxA
		142 (80), **170** (100), 171 (13), 188 (46)			
3	16.1	50 (19, **M**^**+**^), 75 (17), 76 (44), 150 (15), 151 (78),	Anthracene-9,10-quinone	xx	x
		152 (78), **180** (100), 181 (14), 208 (98), 209 (15)			
4	11.8	63 (3, **M**^**+**^), 82 (7), 139 (4), 163 (7), 164 (6),	Anthrone	x	xx
		165 (53),166 (10),193 (9), **194** (100), 195 (15)			
5	11.1	50 (11, **M**^**+**^), 63 (20), 64 (14), 89 (11), 90 (12),	Chromone	x	xxxA
		92 (40), 118 (62), 120 (24), **146** (100), 147(10)			

M = metabolite

Ecotoxicity tests are rapid tests commonly used to determine the impact of contaminants on living organisms (Clasen and Lisbôa 2019). The obtained data on ecotoxicity revealed that the formation of one or more metabolites could induce acute toxicity despite a significant reduction in parent compound concentration. A similar observation was reported where biodegradation of bisphenol by fungi produces metabolites of higher toxicity than the parent compound (Mtibaà et al. 2018). Since quinone derivatives such as chromones are known toxicity inducers which act as a direct-acting mutagen, this could explain the low survival of *V. parahaemolyticus* (log CFU/mL) when exposed to anthracene degradation product of *Tp*FLU12L-mediator (Fig. 5B) within 6 h degradation period (Chibwe et al. 2017; Menzie et al. 1992). Also, the slight reduction in *V. parahaemolyticus* survival (log CFU/mL) as the primary decomposer in the ecosystem and the percentage cell viability of HT-22 cell line as a tertiary consumer could be attributed to the presence of 9-oxo-9*H*-fluorene-1-carboxylic acid. This observation corroborate previous finding which demonstrated that carboxylation on C-1 of fluorene which yielded fluorene-1-carboxylic acid as a metabolite increased acute toxicity in zebrafish than its parent compound (fluorene) (Kim et al. 2020). It is worth noting that the variation in log CFU/mL of the primary decomposer and the tertiary consumer percentage cell viability per each PAHs degradation product has revealed that toxicity is not a function of contaminant concentrations but its molecular structure and exposure time-dependent (Lukić et al. 2016). Also, the most probable explanation for the observed cell viability exceeding 100% in the ABTS degradation products exposed cells further attests to the non-toxic nature of these degradation products with the possibility of the cell line utilizing one or more metabolites, leading to the enhanced metabolic activity of the cell line. This phenomenon is well-documented in various studies, such as those investigating the effects of X-ray irradiation on human lens epithelial cells (Dehankar et al. 2015), the viability of human melanoma cells following drug and proton irradiation treatments (Petrović et al. 2007), and the relationship between cell viability and treatment outcomes in acute myeloid leukaemia (Maha et al. 2008) where no toxic product increased cell viability. Furthermore, a review (Stoddart 2011) highlighted that cell viability could exceed 100% under certain circumstances, often due to enhanced metabolic activities resulting from cell line treatments. This situation commonly arises when calculating the ratio between treated and non-treated cells' optical density (OD) values. For example, if the OD value of treated cells is 0.9 and the OD value of non-treated cells is 0.8, then the cell viability percentage is calculated as 0.9/0.8 × 100 = 112.5%. This indicates an increase in metabolic activity or cell numbers in treated cells compared to non-treated cells.

There is a relatively limited number of existing studies that utilize transcriptomic analysis to investigate the toxicity of metabolic products of polycyclic aromatic hydrocarbons (PAHs). This scarcity of research has prompted us to employ HT-22 cells as a model system to gain further insights into the ecological implications of fluoranthene degradation products. This decision was made based on the low survival rate of bacteria observed and the perceived decrease in cell viability. The observed increase in the expression level of BACE-1, ADAM-10, TAU, PPARγ, and APP genes in the positive control group (uncatalyzed, anthracene only), as well as in cells treated with *Tl*FLU1L degradation product, suggests that these products are neurotoxic and capable of inducing neurodegeneration like Alzheimer’s disease (AD). This observation lends credence to the previous report where these genes were attributed to the production and aggregation of amyloid beta peptides and neurofibrillary tangles, which are the primary causes of neuronal damage and death in AD (Hardy and Selkoe 2002). However, the degradation products of *Tp*FLU12L, *Tl*FLU1L + ABTS, and *Tp*FLU12L + ABTS showed decreased expression of these genes, indicating lower neurotoxicity and a potential for neuroprotection (Fig. 8). These compounds might prevent or reduce the formation and accumulation of amyloid beta peptides and neurofibrillary tangles by modulating the activity or expression of BACE-1 and ADAM-10, which are critical enzymes in the processing of amyloid precursor protein (APP) (Vassar et al. 2014). Also, the exhibited healthy morphological characteristics such as neuronal-like appearance with elongated, spindle-shaped bodies, well-defined nuclei, and uniform neurite processes (Fig. 7C, D) further attest to the lower neurotoxicity of the degradation product from *Tp*FLU12L, *Tl*FLU1L + ABTS, and *Tp*FLU12L + ABTS. The exhibited cytotoxic effects with cellular shrinkage, increased membrane blebbing, and loss of neurite extensions in HT-22 treated cells with positive control (anthracene only) and anthracene oxidation products from *Tl*FLU1L and *Tp*FLU12L can be linked to the neurodegeneration potential of these products due to decrease in cell viability because of swelling and decrease in the microvilli content, varying degrees damages to the cellular membrane integrity, sparse chromatin, very wide gap mitochondria ridge gap and scarcity of the microfilament and microtubule density (Chen et al. 2023).

## Conclusion

This study explored the potential of Laccases from *Tl*FLU1 (*Tl*FLU1L) and *Tp*FLU12L for the biodegradation of anthracene. Our findings demonstrate the superior efficiency of *Tl*FLU1L, particularly when coupled with a Laccase-mediator system. This combined approach enhanced degradation and displayed greater stability compared to the free Laccase system. However, GC–MS analysis revealed a crucial finding: the degradation pathways differed between methods. The mediator system produced benzoic acid through a potentially less harmful route involving hydroxylation and carboxylation, while the free Laccase system utilized a dioxygenation pathway. Significantly, ecotoxicity tests suggest that some degradation products might be more toxic than anthracene. This underscores the importance of evaluating not only degradation efficiency but also the environmental and health impacts of the resulting metabolites. Overall, this study establishes *Tl*FLU1L with its Laccase-mediator system as a promising candidate for eco-friendly bioremediation of anthracene-contaminated environments. The observed differences in degradation pathways highlight the need for further research to fully understand the enzymatic mechanisms and the fate of these metabolites in real-world ecosystems. Additionally, in-vivo studies are crucial to assess the potential risks and benefits of these Laccases for practical environmental clean-up applications. By elucidating the structure–activity relationship of the degradation products, we can develop more sustainable strategies that maximize contaminant removal while minimizing the generation of harmful byproducts. This knowledge is essential for ensuring the safe and effective implementation of enzymatic bioremediation in the field.

### Supplementary Information

Below is the link to the electronic supplementary material.

Supplementary file1 (PDF 152 kb)

## Data Availability

Data are contained within the article or Supplementary Material.
