# Supplementary material for: Anthracene detoxification by Laccases from indigenous fungal strains Trichoderma lixii FLU1 and Talaromyces pinophilus FLU12
Source: Biodegradation. 2024 Jun 1;35(5):769–87. doi: 10.1007/s10532-024-10084-3 (PMC11246312; doi:10.1007/s10532-024-10084-3)
Supplement: Supplementary file 1 — Supplementary file1 (PDF 152 kb) [file 10532_2024_10084_MOESM1_ESM.pdf]

SUPPLEMENTARY MATERIALS

Anthracene Detoxification by Laccases from Indigenous strains *Trichoderma lixii* FLU1 and *Talaromyces pinophilus* FLU12

Samson O. Egbewale[1], Ajit Kumar[1], Tosin A. Olasehinde[a], Mduduzi P. Mokoena[1,2] and Ademola O. Olaniran [1,*]

[1]Discipline of Microbiology, University of KwaZulu-Natal (Westville Campus), Durban-4001, South Africa

[2]Department of Pathology, School of Medicine, University of Limpopo, Private Bag X1106, Sovenga-0727, South Africa.

*Correspondence: olanirana@ukzn.ac.za; Tel.: +27-31-260-7400

Table 1. The composition of the BSM media

Component	Amount (g/L)	Trace Elements	Amount (mg/L)
$(NH_4)_2SO_4$	2.4	Nitrilotriacetic acid	15
K_2HPO_4	1.55	$CaCl_2.2H_2O$	15
$NaH_2PO_4.2H_2O$	0.85	$MnCl_2.2H_2O$	6
NaCl	0.5	$FeSO_4.7H_2O$	1
$MgSO_4.7H_2O$	0.26	$Co(NO_3).6H_2O$	1
		$ZnSO_4$	1
		$CuSO_4$	0.1
		$H3BO_3$	0.1
		Na_2MoO_4	0.1
		$Al_2(SO_4)_3.H_2O$	0.1

Table S2. The percentage of residual Anthracene concentration after the degradation by *Tl*FLU1L and *Tp*FLU12L (2U).

Time (h)	Control	*Tl*FLU-1	*Tp*FLU-12
0	99.9±0.2	99.9±0.2	99.9±0.2
24	99.9±0.2	73.9±5.1	83.1±7.2
48	99.9±0.2	60.1±1.3	77.3±8.4
72	99.9±0.2	52.1±6.1	62.0±3.1
96	99.9±0.2	44.9±3.0	50.1±9.2

Table S3. The influence of *Tl*FLU1L and *Tp*FLU12L concentrations on *in vitro* degradation of anthracene (96 h).

U/mL	*Tl*FLU-1	*Tp*FLU-12
0	99.9±0.2	99.9±0.2
3	44.3±8.1	42.5±4.1
6	36.5±3.1	19.3±8.8
8	28.7±8.5	3.9±3.1
10	nd	nd

nd=not detected

Table S4. The influence of mediator (ABTS) concentration on *invitro* oxidation of anthracene by *Tl*FLU1L (2U).

**Time (h)**	**0 mM**	**200 µM**	**500 µM**	**1 mM**	**5 mM**	**10 mM**
0	99.9±0.2	99.9±0.2	99.9±0.2	99.9±0.2	99.9±0.2	99.9±0.2
3	96.1±7.2	55±1.7	53.9±4	49.6±7	48.8±6	44.3±5
6	88.1±2.1	47.3±3	46.9±3	44.9±2	41.7±5	38±2
12	80.1±3.3	11.4±3	6.9±3	6.6±3	nd	nd
24	72.5±5.1	6.1±2	5.9±5	4.3±3	nd	nd
48	64.5±4.3	nd	nd	nd	nd	nd
72	56.6±6.4	nd	nd	nd	nd	nd
96	44.3±1.5	nd	nd	nd	nd	nd

nd=not detected

Table S5. The influence of mediator (ABTS) concentration on *invitro* oxidation of anthracene by *Tp*FLU12L (2U).

**Time (h)**	**0 mM**	**200 µM**	**500 µM**	**1 mM**	**5 mM**	**10 mM**
0	99.9±0.2	99.9±0.2	99.9±0.2	99.9±0.2	99.9±0.2	99.9±0.2
3	96.8±4.2	57.1±5.6	56.8±1	55.3±3.3	53.9±2.5	53.4±5.4
6	93.6±5.8	50.2±4.6	49.1±7.5	48.8±1.2	48.2±1.7	47.2±6
12	84±3.5	10.3±5.5	9.3±3.2	8.7±6.4	8.4±7.2	7.3±5.4
24	71.3±3.7	8.5±3.9	8.8±1.7	6.6±2	4.1±1.2	6±3.8
48	64.9±2.2	nd	nd	nd	nd	nd
72	48.9±9.2	nd	nd	nd	nd	nd
96	42.5±3.1	nd	nd	nd	nd	nd

nd=not detected
